# Oxytocin Reduces Intravesical Pressure in Anesthetized Female Rats: Action on Oxytocin Receptors of the Urinary Bladder

**DOI:** 10.3389/fphys.2020.00382

**Published:** 2020-05-06

**Authors:** Eduardo M. Cafarchio, Luiz A. da Silva, Luciana C. Auresco, Itatiana F. Rodart, Janaina S. de Souza, Bruno B. Antonio, Daniel P. Venancio, Laura B. M. Maifrino, Rui M. B. Maciel, Gisele Giannocco, Patrik Aronsson, Monica A. Sato

**Affiliations:** ^1^Department Morphology and Physiology, Faculdade de Medicina do ABC, Centro Universitário Saúde ABC, Santo André, Brazil; ^2^Department Collective Health, Human Reproduction and Genetics Center, Faculdade de Medicina do ABC, Centro Universitário Saúde ABC, Santo André, Brazil; ^3^Department Medicine, Federal University of São Paulo, São Paulo, Brazil; ^4^Laboratory of Histomophometry, Universidade São Judas Tadeu, São Paulo, Brazil; ^5^Department Pharmacology, Institute of Neuroscience and Physiology, Sahlgrenska Academy, University of Gothenburg, Gothenburg, Sweden

**Keywords:** intravesical pressure, urinary bladder, oxytocin, receptors, vasopressin (ADH)

## Abstract

Urinary bladder dysfunction affects several people worldwide and shows higher prevalence in women. Micturition is dependent on the Barrington’s nucleus, pontine urine storage center and periaqueductal gray matter, but other brain stem areas are involved in the bladder regulation. Neurons in the medulla oblongata send projections to hypothalamic nuclei as the supraoptic nucleus, which synthetizes oxytocin and in its turn, this peptide is released in the circulation. We investigated the effects of intravenous injection of oxytocin (OT) on the urinary bladder in sham and ovariectomized rats. We also evaluated the topical (*in situ)* action of OT on intravesical pressure (IP) as well as the existence of oxytocin receptors in the urinary bladder. In sham female Wistar rats, anesthetized with isoflurane, intravenous infusion of OT (10 ng/kg) significantly decreased the IP (–47.5 ± 1.2%) compared to saline (3.4 ± 0.7%). Similar effect in IP was observed in ovariectomized rats after i.v. OT (–41.9 ± 2.9%) compared to saline (0.5 ± 0.6%). Topical administration (*in situ*) of 0.1 mL of OT (1.0 ng/mL) significantly reduced the IP (22.3.0 ± 0.6%) compared to saline (0.9 ± 0.7%). We also found by qPCR that the gene expression of oxytocin receptor is present in this tissue. Blockade of oxytocin receptors significantly attenuated the reduction in IP evoked by oxytocin i.v. or *in situ*. Therefore, the findings suggest that (1) intravenous oxytocin decreases IP due to bladder relaxation and (2) OT has local bladder effect, binding directly in receptors located in the bladder.

## Introduction

The bladder dysfunctions cause social and mental discomfort and affect the well-being due to the difficulty of performing several normal activities in daily life. Dysfunctions of the lower urinary tract are frequent complaints, accounting for up to 40% in ambulatories of nephrology and urology (Bakker et al., 2002; Kajiwara et al., 2004; Hashim et al., 2009; Sureshkumar et al., 2009). The urinary bladder disorders, particularly urinary incontinence symptoms show higher prevalence in women (Aoki et al., 2017).

The detrusor of the urinary bladder is composed of smooth muscle with gap junctions between its cells (Steers, 1998). The detrusor and the internal sphincter are innervated by the autonomic nervous system. Urine storage and micturition depend on coordination between two functional units: the urinary bladder and the striated musculature of the urethral sphincter (de Groat, 1998; Andersson and Hedlund, 2002).

Albeit complex, it is known that the central control of micturition is dependent on Barrington’s nucleus (pontine micturition center), pontine urine storage center (PUSC) and periaqueductal gray matter (PAG) (de Groat, 1998). Brain stem areas such as the nucleus of the solitary tract (NTS), caudal ventrolateral medulla (CVLM), and rostral ventrolateral medulla (RVLM) are best known for their involvement in cardiovascular regulation. Nevertheless, it has also been shown that they can elicit changes in pelvic nerve activity (Chen and Chai, 2002; Cafarchio et al., 2018). Previous studies have demonstrated that the NTS and RLVM have projections for hypothalamic nuclei, which contains the cell bodies of neurons responsible for the production of vasopressin and oxytocin (OT). Those peptides are transported through the axons and released by the neurophypophysis in the circulation (Ross et al., 1984; Romine and Anderson, 1985). While those areas can elicit vasopressin release by cholinergic activation of medullary neurons, activation or (Cafarchio et al., 2016) blockade of cholinergic receptors in the medulla does not release OT in the plasma (Cafarchio et al., 2016). On the other hand, OT neurons in the supraoptic nucleus receive noradrenergic projections from the A1 and A2 cell groups in the medulla oblongata (Onaka et al., 2001; Zhu and Onaka, 2002). The A2 noradrenergic neurons seem to be involved in the activation of OT neurons after conditioned fear stimuli (Zhu and Onaka, 2002), whereas A1 noradrenergic neurons may mediate OT release after noxious stimuli (Onaka et al., 2001).

Oxytocin is a nonapeptide synthesized in the paraventricular nucleus (PVN) and supraoptic nucleus (SON), which are both hypothalamic nuclei. Magnocellular neurons located in the PVN and SON are responsible for the largest part of the OT release but a small percentage of OT is produced by parvocellular neurons of the PVN (Gutkowska et al., 2000a, b; Stern, 2015). The hormone is transported by carrier proteins from the magnocellular neurons of the SON and PVN and parvocellular neurons of the PVN to the neurohypophysis, where the neuropeptide is stored and released into the bloodstream (Swanson and Sawchenko, 1980; Johnson and Thunhorst, 1997; Freeman and Young, 2016). Oxytocin is mostly known for its peripheral action in milk ejection and parturition (Romine and Anderson, 1985; Onaka et al., 2001), however studies on isolated preparations have shown that oxytocin elicits contraction of the rabbit detrusor muscle (Romine and Anderson, 1985). Oxytocin also has a protective role during ischemia and reperfusion injury in the rat urinary bladder (Du Vigneaud, 1954). Nevertheless, to the best of our knowledge the existence of oxytocin receptors has not been demonstrated in the urinary bladder.

The ovarian hormone estrogen is an important inducer of OT receptors in several tissues (Bale et al., 1995; Zigon, 2013; Narita et al., 2016) and exerts physiological functions, such as in parturition and milk ejection, through the release of OT (Zingg et al., 1995; Zhou and Forsling, 2000; Narita and Ichimaru, 2014). Evidence also indicates that the effects of OT on the magnitude of diuresis and natriuresis are dependent on the phase of the estrous cycle (Zhou and Forsling, 2000).

No previous study has described the existence neither functionality of OT receptors in the rat urinary bladder nor the effects of systemic and topical OT administration on the bladder in anesthetized rats. Thereby, this study focused to investigate the effects of intravenous injection of OT on the urinary bladder in intact and ovariectomized female rats. We also evaluated the topical (*in situ)* action of OT on intravesical pressure (IP) as well as the expression of oxytocin receptors in the urinary bladder.

## Materials and Methods

### Animals

Thirty-two female Wistar rats (∼250–300 g, 14–16 weeks-old) provided by the Animal Facility of the Faculdade de Medicina do ABC were used. The animals were housed in groups of 3 rats/cage, except when they were submitted to ovariectomy or sham surgery, in which they were maintained in individual plastic cages until healing. Animals had access to standard chow pellets and tap water *ad libitum*, and were maintained in an air conditioned room (20–24°C) with a 12:12 h light-dark cycle. The humidity of the animal room was set at ∼70%. All procedures were performed in accordance with the National Institutes of Health (NIH) Guide for the Care and Use of Laboratory Animals, and were approved by the Animal Ethics Committee of the Faculdade de Medicina do ABC (protocol number 007/2011).

### Ovariectomy Surgery

The group of ovariectomized rats was anesthetized with ketamine (50 mg/kg, i.p., Dopalen^®^, Ceva Saude Animal, Paulinia, Brazil) and xylazine (10 mg/kg, i.m, Anasedan^®^, Ceva Saude Animal, Paulinia, Brazil). After abdominal trichotomy, an incision of about 2 cm was made in the lateral flank until reaching the abdominal cavity. The ovaries were exposed, tied and completely harvested bilaterally and the incisions were subsequently suture closed. At the end of surgery, the rats received a single dose of Veterinary Pentabiotic for Small Animals (2,000 U/mL, i.m., FortDodge, Campinas, Brazil) as a prophylactic procedure and tramadol (10 mg/kg, i.m., Tramal^®^, Pfizer, Sao Paulo, Brazil) at the end of surgery and every 12 h in the immediate post-operative period (1 day after surgery). The animals recovered from surgery for 21 days in order to reduce the circulating estrogen levels, in accordance to earlier studies (Deer and Stallone, 2016). Sham rats underwent the same surgical procedures, but the ovaries were not removed.

### Cannulation of the Urinary Bladder

The rats were anesthetized with 2% isoflurane (BioChimico, Penedo, Itatiaia, Brazil) in 100% O_2_ and subjected to a partial laparotomy and a small incision in the bladder wall was performed for insertion of a polyethylene tubing (PE-50 connected to PE-10, Clay Adams, NJ, United States) filled with saline at the appex of the bladder. A small drop of tissue glue was used to fix the catheter on the bladder wall for IP recordings in a data acquisition system (PowerLab 16 SP, AD Instruments, Castle Hill, AU, United States). The abdominal cavity was covered with gaze dressing humidified with saline. The urethra outlet was not submitted to ligature in order to permit the bladder voiding if necessary. A baseline IP value was set at ∼8–10 mmHg by saline infusion or urine withdrawal through the catheter inserted into the urinary bladder.

### Cannulation of the Femoral Artery and Vein

The rats anesthetized with 2% isoflurane (BioChimico, Penedo, Itatiaia, RJ, Brazil) in 100% O_2_ were subjected to cannulation of the femoral artery and vein by inserting a polyethylene tubing (PE-50 connected to PE-10, Clay Adams, NJ, United States) for pulsatile arterial pressure (PAP), mean arterial pressure (MAP) and heart rate (HR) recordings in the data acquisition system (PowerLab 16 SP, AD Instruments, Castle Hill, AU, United States), and for drug administration, respectively.

### RNA Isolation and Quantitative Real-Time RT-PCR

Total RNA was isolated from frozen urinary bladder (weighing ∼100 mg) with TRIzol Reagent^®^ (Life Technologies Corporation, Carlsbad, CA, United States) according to the manufacturer’s protocol. RNA integrity was checked by agarose gel electrophoresis, and RNA purity reached the following criteria: A260/280 ≥ 1.8. The extracted total RNA concentration was measured using a Nanodrop spectrophotometer (ND-1000) (Bio-Rad, United States), and 1 μg of total RNA was subjected to reverse transcription reaction. Complementary DNA (cDNA) synthesis was generated using ImProm-II^TM^ Reverse Transcription System (Promega, Madison, WC) according to the manufacturer’s protocol. Quantitative real-time PCR (qPCR) was carried out using 2 μL of cDNA and the SYBR^TM^ Green PCR Master Mix (Thermo Fisher Scientific, Waltham, MA, United States) in the ABI Prism 7500 Sequence Detection System (Applied Biosystems, Foster City, CA, United States) to amplify specific primers sequences for oxytocin receptors. The forward and reverse primers sequences for rats were, respectively:

OT receptor: (forward)- 5′-CATGCTGCTGGCTAGCCTTA-3′

            (reverse)- 5′-CAAAGCAGGCTACGCAACTC-3′

Cyclophilin A: (forward)- 5′-CCCACCGTGTTCTTCGACAT-3′;

            (reverse)- 5′-CTGTCTTTGGAACTTTGTCTGCAA-3′

Cyclophilin A was used as internal control (housekeeping gene). The procedure consisted of an initial step of 10 min at 95°C followed by 45 cycles of 20 s each at 95°C, 20 s at 58°C, and 20 s at 72°C. Gene expression was determined by CT, and all values were expressed using cyclophilin A mRNA as an internal control.

### Experimental Protocols

#### Intravenous Injection of Oxytocin on Urinary Bladder and Cardiovascular Parameters in Sham and Ovariectomized Rats

Sham (control) and ovariectomized rats were anesthetized with isoflurane and submitted to catheterization of the femoral artery and vein, and cannulation of the urinary bladder as described above. After the baseline recordings of PAP, MAP, HR, and IP for 15 min, saline (1 mL/kg, vehicle) or oxytocin (oxytocin acetate salt hydrate, Sigma Aldrich cat#O6379, lot# EC 200-048-4, St. Louis, MO, United States) at 1.0 ng/kg of b.w. was administrated for 5 min using an infusion pump (Insight Ltda, Ribeirão Preto, SP, Brazil). Saline or oxytocin were randomly injected in the animals. The oxytocin solution was prepared in a concentration of 1.0 ng/mL. All the parameters were recorded during the infusion and for additional 30 min. Each infusion was carried out only after the parameters have recovered to baseline levels. At the end of the experiments, rats were euthanized with an overdose of sodium thiopental (70 mg/kg, i.v.).

#### Effects of *in situ* Oxytocin Administration on the Urinary Bladder and Cardiovascular Parameters in Sham Rats

Under isoflurane anesthesia, rats underwent to the catheterization of the femoral artery and cannulation of the urinary bladder. After the baseline recording of PAP, MAP, HR, and IP for 15 min, 0.1 mL of saline or oxytocin (oxytocin acetate salt hydrate, Sigma Aldrich cat#O6379, lot# EC 200-048-4, St. Louis, MO, United States) (1.0 ng/rat) was dropped on the outside of the urinary bladder, in order to evaluate the topical (*in situ*) action. All animals received both saline and oxytocin topically on the urinary bladder and the parameters were recorded for 30 min. Each *in situ* administration was carried out only after the parameters had recovered to baseline levels. At the end of the experiments, rats were euthanized with an overdose of sodium thiopental (70 mg/kg, i.v.).

#### Intravenous and *in situ* Oxytocin Effects on Urinary Bladder and Cardiovascular Parameters Upon Blockade of Oxytocin Receptors in Sham Rats

Sham (control) rats were anesthetized with isoflurane and submitted to catheterization of the femoral artery and vein, and cannulation of the urinary bladder as described above. After the baseline recordings of PAP, MAP, HR, and IP for 15 min, β-Mercapto-β,β-cyclopentamethylenepropionyl^1^, O-Me-Tyr^2^, Orn^8^]-Oxytocin (10 μg/kg of b.w, Sigma Aldrich cat# O6887 lot#079K1684, St. Louis, MO, United States), an oxytocin receptor antagonist, was administrated intravenously for 5 min using an infusion pump (Insight Ltda, Ribeirão Preto, Brazil). After 30 min, oxytocin 1 ng/kg was injected intravenously or *in situ* (0.1 ng/rat) onto the bladder surface in the animals. The oxytocin solution was prepared in a concentration of 1.0 ng/mL. All the parameters were recorded during the infusion and for additional 30 min. At the end of the experiments, rats were euthanized with an overdose of sodium thiopental (70 mg/kg, i.v.).

#### Gene Expression of Oxytocin Receptor on the Urinary Bladder

In a separate group of animals (*N* = 6), which was different from those included in the functional experiments of this study, rats were deeply anesthetized with 2% isoflurane in 100% O_2_ and a midline incision was performed in the abdomen and the urinary bladder was harvested and immediately frozen in liquid nitrogen. Afterward, the samples were stored in an ultrafreezer at –80°C until RNA extraction. All the animals used for gene expression were intact rats. The further procedures for gene expression of oxytocin receptor in the urinary bladder were performed by qPCR, according to the procedures described in section RNA Isolation and Quantitative Real-Time RT-PCR. After the bladder was harvest, the rats were euthanized with an overdose of sodium thiopental (70 mg/kg, i.v.).

### Statistics

A Komolgorov-Smirnov test for normality was used for verifying the data distribution. Once the results fit to a normal distribution, they were expressed as mean ± S.E.M. Data were submitted to paired Student *t*-test for comparison between the baseline and the responses after i.v. or topical oxytocin intra-group, and unpaired Student *t*-test for comparison of baseline or the responses after oxytocin between sham and ovariectomized rats. Statistical analysis were conducted using the statistical software package Sigma Stat 3.5. Significance level was set at *P* < 0.05.

## Results

### Effects of Intravenous Infusion of Oxytocin on Intravesical Pressure, Arterial Pressure, and Heart Rate in Sham and Ovariectomized Rats

At baseline (before intravenous injection of oxytocin), the MAP was 92 ± 5 mmHg, the HR was 300 ± 14 bpm and the IP was 11 ± 1.0 mmHg in sham (control) rats (*N* = 7).

**FIGURE 1:**
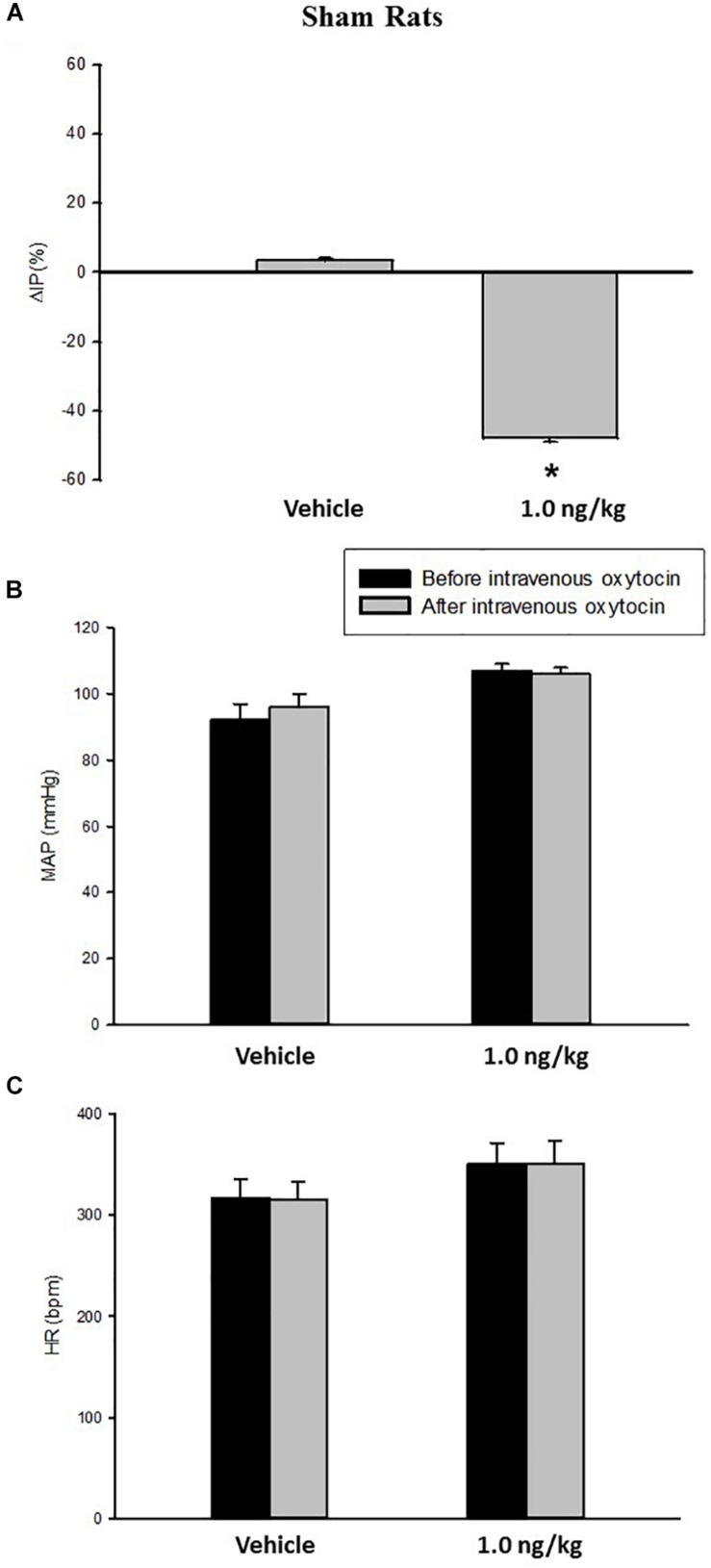
Percent change in intravesical pressure (**A**, %ΔIP) after intravenous injection of saline (vehicle) or oxytocin dose (1.0 ng/kg of b.w.) in sham animals. Mean arterial pressure (**B**, MAP, mmHg) and heart rate (**C**, HR, bpm) at baseline and after intravenous injection of saline (vehicle) or oxytocin dose (1.0 ng/kg of b.w.) in sham animals, *p < 0.05 vs. saline (*N* = 7).

Intravenous infusion of oxytocin 1.0 ng/kg in sham rats evoked a significant decrease in IP (–47.5 ± 1.2%) compared to saline infusion (3.4 ± 0.7%) (Figures 1, 3). The onset of the response in IP occurred at 10 min after starting the oxytocin infusion and the peak response was achieved at 15 min after the onset of infusion. The fall in IP lasted for additional 5 min after achieving the peak response.

No significant changes were observed in MAP, and HR after the injection of oxytocin in sham rats (Figures 1, 3).

In ovariectomized rats, at baseline (before the intravenous injection of oxytocin) the MAP was 97 ± 1 mmHg, the HR was 295 ± 19 bpm and the IP was 12 ± 0.5 mmHg (*N* = 6).

**FIGURE 2:**
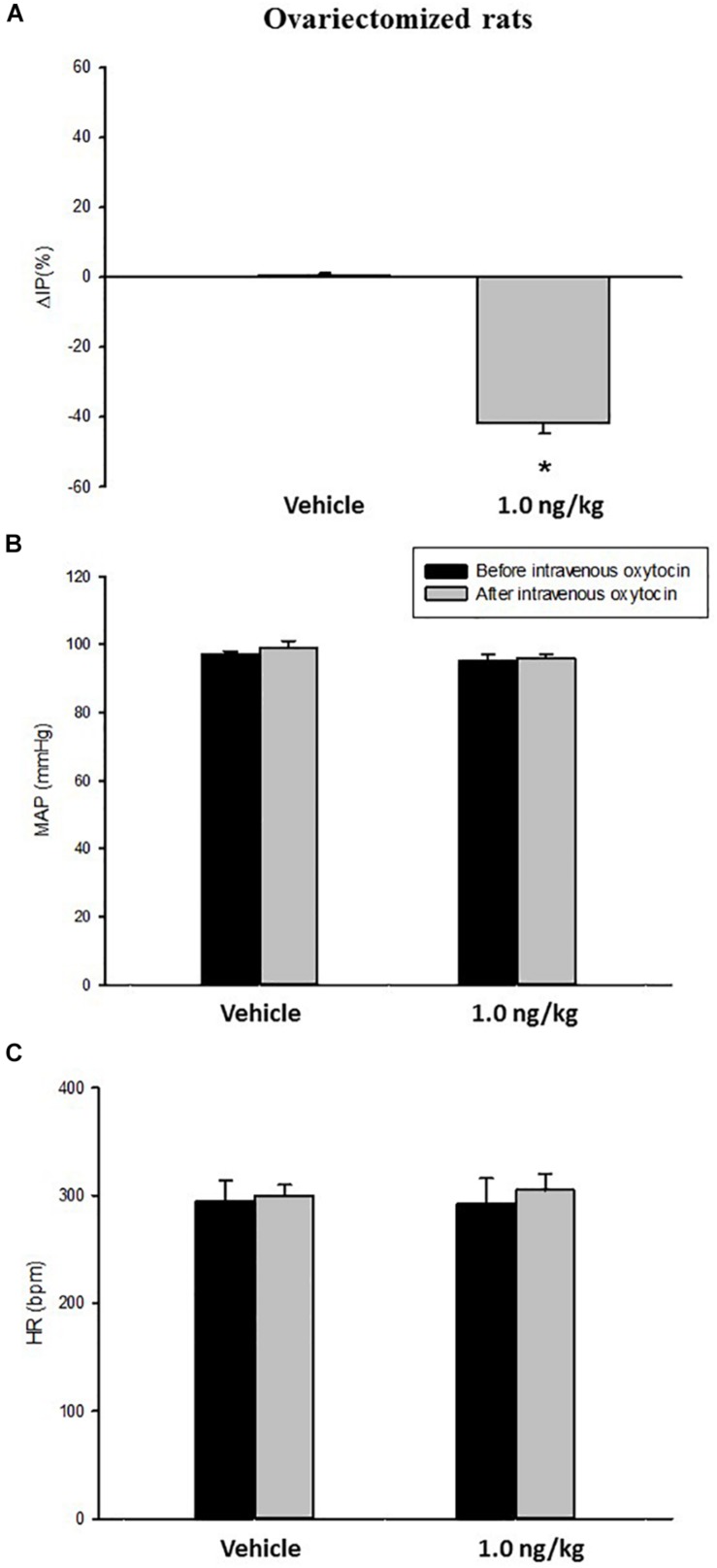
Percent change in intravesical pressure (**A**, % ΔIP) after intravenous injection of saline (vehicle) or oxytocin dose (1.0 ng/kg of b.w.). Mean arterial pressure (**B**, MAP, mmHg) and heart rate (**C**, HR, bpm) at baseline and after intravenous injection of saline (vehicle) or oxytocin dose (1.0 ng/kg of b.w.) in ovariectomized animals *p < 0.05 vs. saline (*N* = 6).

**FIGURE 3:**
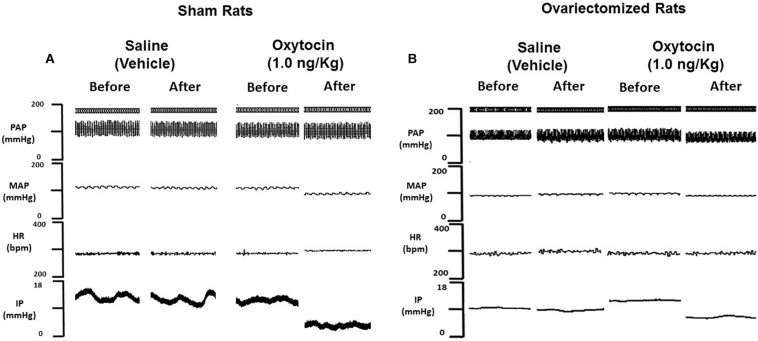
Tracings showing pulsatile arterial pressure (PAP, mmHg), mean arterial pressure (MAP, mmHg), heart rate (HR, bpm) and intravesical pressure (IP, mmHg) before and after intravenous infusion of saline (vehicle) or oxytocin 10 ng/kg in sham **(A)** and ovariectomized **(B)** rats.

Intravenous infusion of oxytocin (1.0 ng/kg) in ovariectomized rats (*N* = 6) elicited a significant reduction in IP (–41.9 ± 2.9%) compared to saline infusion (0.5 ± 0.6%) (Figures 2, 3). Similarly to the sham (control) group, the onset of the response was 10 min after the beginning of oxytocin infusion and the peak response was achieved at 15 min after starting the infusion of oxytocin. The decrease in IP lasted for additional 5 min after achieving the peak response.

No significant changes were observed in MAP and HR after the infusion of oxytocin in ovariectomized rats (Figures 2, 3).

We observed no difference in the IP and cardiovascular responses evoked by i.v. oxytocin comparing ovariectomized and sham rats.

### Effects of the *in situ* Administration of Oxytocin on the Intravesical Pressure and Cardiovascular Parameters in Sham Rats

At the baseline (before *in situ* administration of oxytocin), rats showed 95 ± 3 mmHg of MAP, 315 ± 22 bpm of HR, and 8.3 ± 1.7 mmHg of IP (*N* = 7).

**FIGURE 4:**
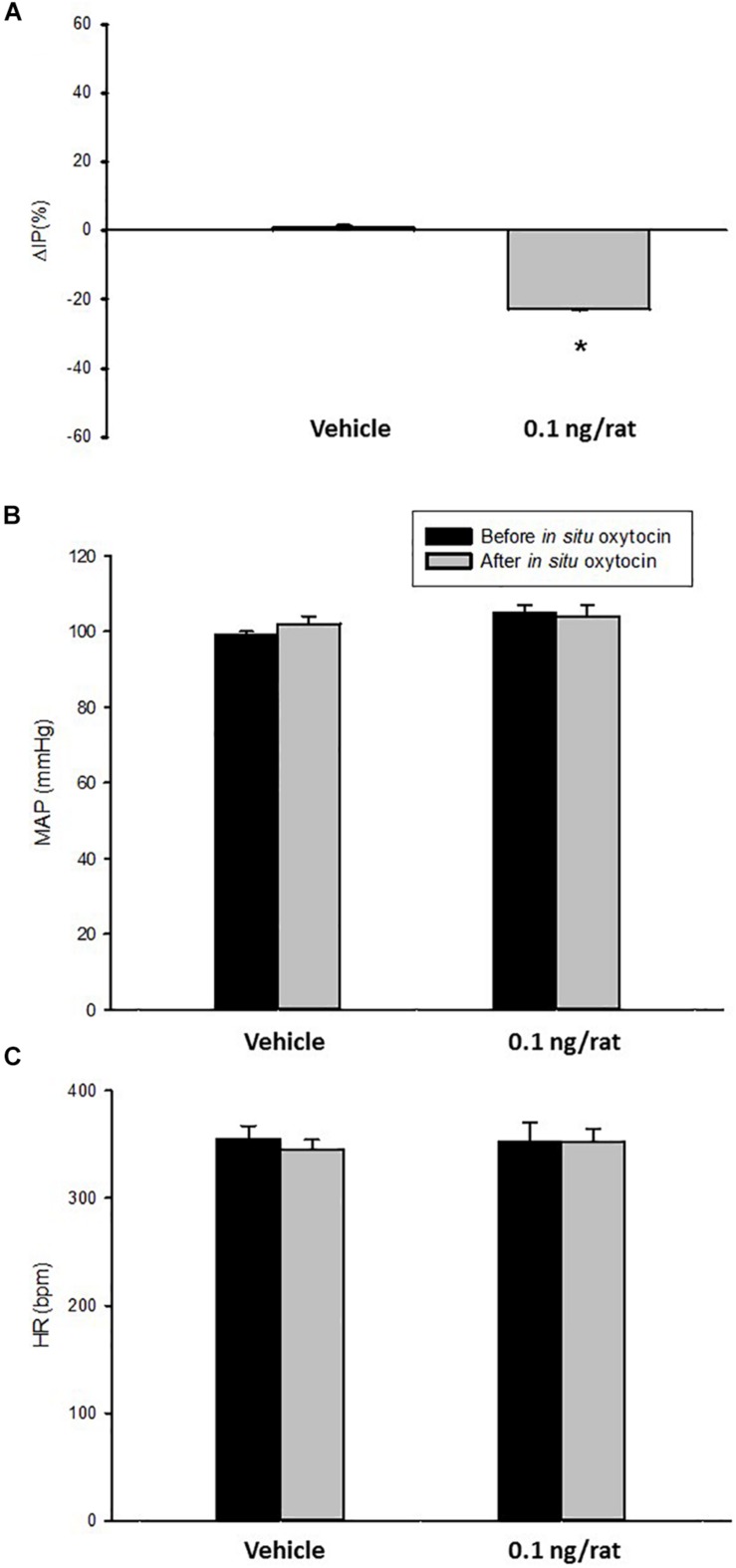
Percent change in intravesical pressure (**A**,% ΔIP) after *in situ* administration of 0.1 mL of saline (vehicle) or oxytocin (0.1 ng/rat) in sham animals (*N* = 7). Mean arterial pressure (**B**, MAP, mmHg) and heart rate (**C**, HR, bpm) at baseline and after *in situ* administration of 0.1 mL of saline (vehicle) or oxytocin dose (0.1 ng/rat) in sham animals, *p < 0.05 vs. saline (*N* = 6).

**FIGURE 5:**
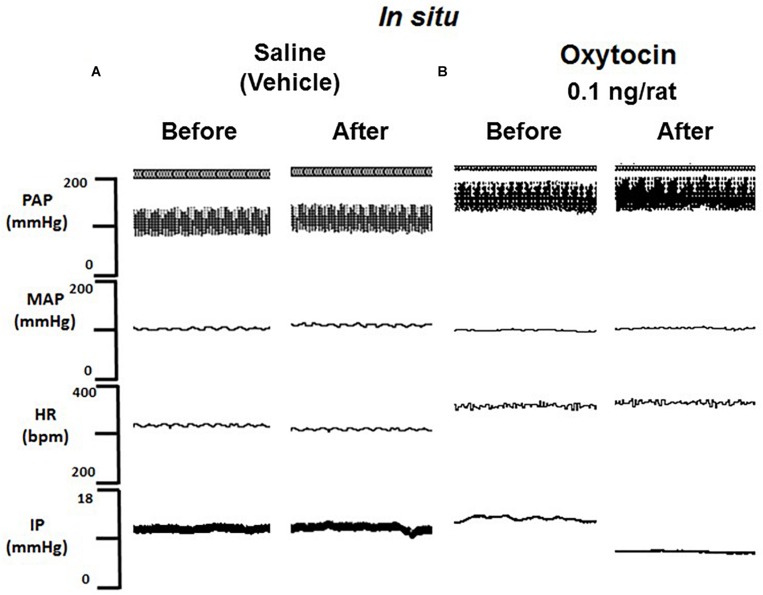
Tracing showing pulsatile arterial pressure (PAP, mmHg), mean arterial pressure (MAP, mmHg), heart rate (HR, bpm) and intravesical pressure (IP, mmHg) before and after saline **(A)** or oxytocin 0.1 ng/rat **(B)** *in situ* administration on the outside of the urinary bladder in sham animals.

Topical administration (*in situ*) of oxytocin (0.1 ng/rat) elicited a significant decrease in IP (–22.3 ± 0.6%) compared to saline administration (0.9 ± 0.7%; Figures 4, 5). The decrease in IP was observed about 5 min after oxytocin was topically dropped onto the bladder. The fall in IP lasted for additional 5 min after achieving the peak response.

No significant changes were observed in MAP and HR after the administration of oxytocin compared to saline (Figures 4, 5).

### Intravenous and *in situ* Oxytocin Effects on Urinary Bladder and Cardiovascular Parameters Upon Blockade of Oxytocin Receptors in Sham Rats

**FIGURE 6:**
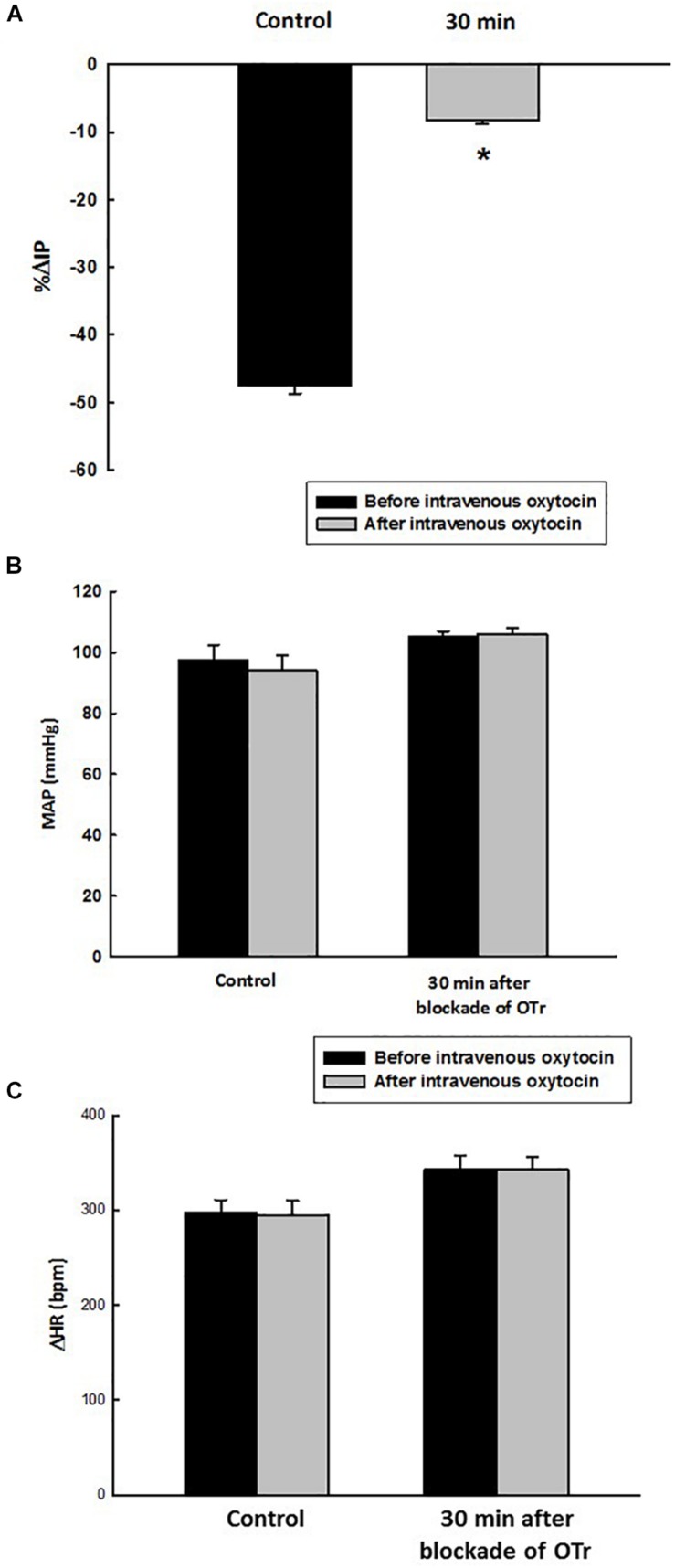
Percent change in intravesical pressure (**A**, % ΔIP) evoked by intravenous injection of oxytocin dose (1.0 ng/kg of b.w., control response) and at 30 min after blockade of oxytocin receptors (OTr) (10 ug/kg, i.v.) in intact rats. Mean arterial pressure (**B**, MAP, mmHg) and heart rate (**C**, HR, bpm) at baseline and after intravenous injection of oxytocin dose (1.0 ng/kg of b.w., control response) and also at 30 min after blockade of oxytocin receptors (10 ug/kg, i.v.) in sham rats, *p < 0.05 vs. control (*N* = 5).

At the baseline (before administration of oxytocin receptor antagonist, OTra), rats of the OTra + i.v. OT group showed 104 ± 2 mmHg of MAP, 368 ± 10 bpm of HR and 10.1 ± 0.3 mmHg of IP (*N* = 5). Thirty minutes after intravenous injection of oxytocin receptor blockade, no significant changes were observed in MAP and HR. IP showed only a small decrease to 9.1 ± 0.2 mmHg. Intravenous injection of oxytocin after intravenous blockade of oxytocin receptors showed a significant attenuated decrease in IP (–8.2 ± 0.6%) compared to the i.v. oxytocin control response in sham rats (–41.9 ± 2.9%) shown in section Effects of Intravenous Infusion of Oxytocin on Intravesical Pressure, Arterial Pressure, and Heart Rate in Sham and Ovariectomized Rats (Figure 6).

**FIGURE 7:**
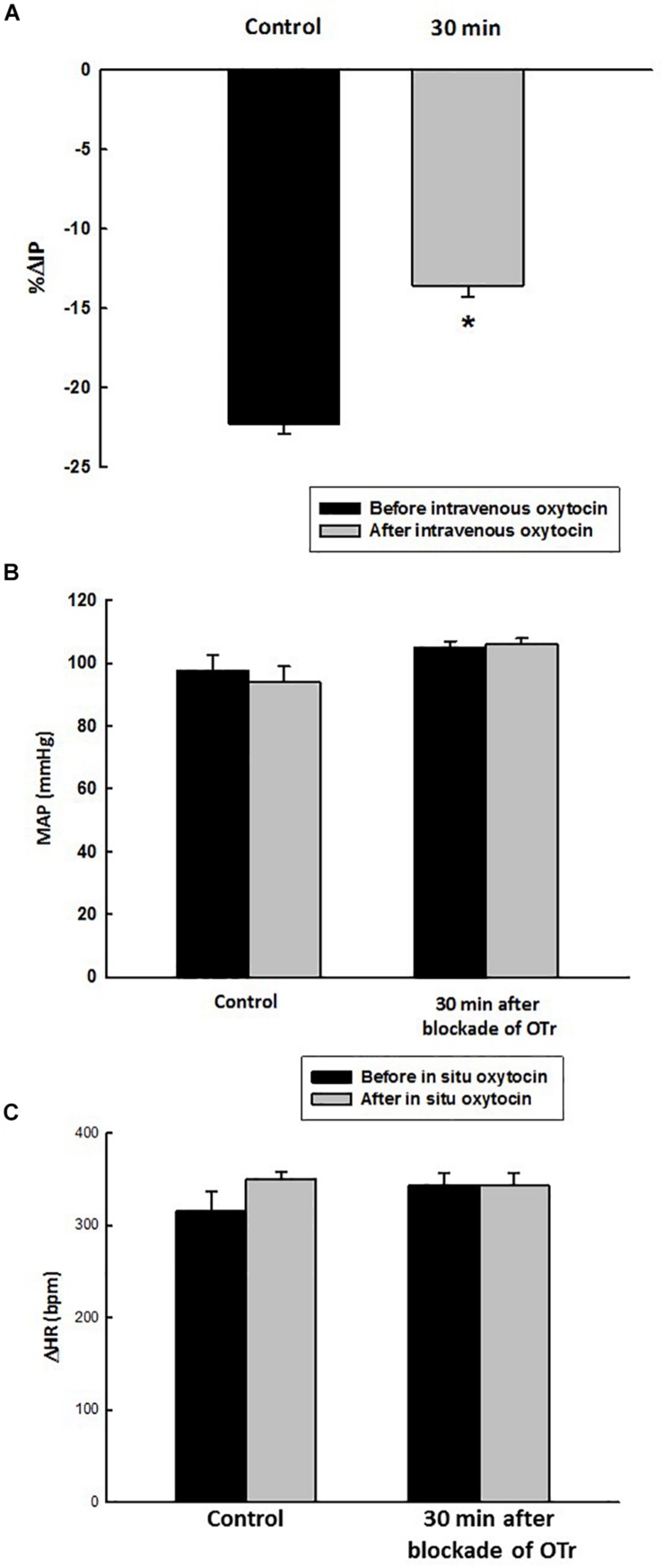
Percent change in intravesical pressure (**A**, % ΔIP) after *in situ* administration of 0.1 mL of saline (vehicle) or oxytocin (0.1 ng/rat) (*N* = 7) at 35 min after blockade of oxytocin receptors (10 ug/kg, i.v.) in intact rats. Mean arterial pressure (**B**, MAP, mmHg) and heart rate (**C**, HR, bpm) at baseline and after *in situ* administration of 0.1 mL of saline (vehicle) or oxytocin dose (0.1 ug/rat mL) at 35 min after blockade of oxytocin receptors (OTr) (10 ug/kg, i.v.) in sham rats, *p < 0.05 vs. control (*N* = 5).

Before administration of oxytocin receptor antagonist (OTra), rats of the OTra + *in situ* OT group at baseline showed 106 ± 2 mmHg of MAP, 358 ± 5 bpm of HR and 9.7 ± 0.8 mmHg of IP (*N* = 5). Thirty minutes after intravenous injection of oxytocin receptor blockade, no significant changes were observed in MAP, HR and IP (9.7 ± 1.1 mmHg). *In situ* oxytocin after intravenous blockade of oxytocin receptors also showed a significant attenuated decrease in IP (–13.6 ± 0.7%) compared to the i.v. oxytocin control response in sham rats (–22.3 ± 0.6%) shown in section Effects of the *in situ* Administration of Oxytocin on the Intravesical Pressure and Cardiovascular Parameters in Sham Rats (Figure 7).

### Gene Expression of Oxytocin Receptor in the Urinary Bladder

**FIGURE 8:**
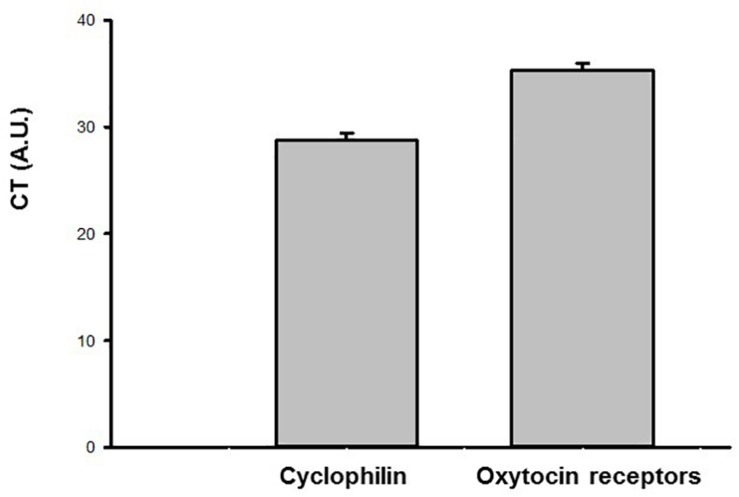
Ct values (arbitrary units, A.U.) obtained by qPCR for the oxytocin (OT) receptor and endogenous housekeeping gene (Cyclophilin A) in the urinary bladder samples of sham animals (*N* = 6).

The results of the gene expression by qPCR in the urinary bladder demonstrated that the oxytocin receptor as well as the housekeeping gene cyclophilin A is present in this tissue (*N* = 6). The CT values for the oxytocin receptor and cyclophilin A are shown in Figure 8.

## Discussion

Our study showed, to the best of our knowledge, for the first time that intravenous administration of oxytocin decreases the IP in anesthetized rats. In sham (control) rats (without ovariectomy), oxytocin did not change any of the cardiovascular parameters studied, suggesting that the reduction in IP is not dependent on filtration pressure changes in the kidneys. Thus, it seems likely that the decrease in IP is independent of any reduction in urinary volume. There is no previous evidence in the literature of oxytocin being a modulator of urinary bladder function. In the current study, intravenous oxytocin elicited an opposite response to that previously shown for i.v. vasopressin (Cafarchio et al., 2018), although in similar concentration (1 ng/mL/kg). Vasopressin increased the contractility of urinary bladder muscle in rabbits, humans and rats at *in vitro* studies (Crankshaw, 1989; Holmquist et al., 1991; Dehpour et al., 1997). In anesthetized female rats, intravenous vasopressin increased the IP in a dose-dependent manner, and the increases were significantly attenuated by blockade of V1a and V2 receptors (Cafarchio et al., 2018). All subtypes of vasopressin receptors (V1a, V1b, and V2) were also found by gene and protein expression in the detrusor muscle (Cafarchio et al., 2018).

Indeed, at least in female rats, oxytocin and vasopressin can bind to their specific receptors and underpin the findings of the current study demonstrating that oxytocin can act in specific receptors in the detrusor muscle to decrease IP.

*In vitro* studies of isolated rabbit bladders have shown that, in contrast to the current study, oxytocin at higher doses increased the contractility of the detrusor muscle (Romine and Anderson, 1985; Pandita et al., 1998). This effect could be explained by the fact that in high doses oxytocin, due to its molecular similarity to vasopressin, can unspecifically bind to, and activate, vasopressin receptors, increasing the urinary bladder contraction in rabbits, humans and rats as shown earlier in isolated preparations (Crankshaw, 1989; Holmquist et al., 1991; Dehpour et al., 1997). Our data showed that the oxytocin receptor gene is present in the urinary bladder, suggesting that oxytocin can decrease the IP through binding in its own receptor in the bladder.

In the current study, we have described the effects of only one dose (1 ng/kg) of oxytocin and we did not include higher doses of oxytocin in order to avoid non-selective actions of oxytocin on other types of receptors as the vasopressin receptors.

Earlier studies of Petty et al. (1985) have shown that intravenous injection of oxytocin (10 ug) decreased mean arterial pressure and heart rate in conscious male rats, however, oxytocin at lower doses (100 pg and 10 ng) evoked no changes in the cardiovascular parameters. In the present study, we have used 1 ng/kg intravenously. Considering that the animals were weighing ∼250 g, each rat has received ∼0.25 ng (or 250 pg or 0.00025 μg) of oxytocin, which were close to the doses that elicited no changes in the cardiovascular parameters as shown in the study of Petty et al. (1985). Therefore, our findings are consistent with previous findings (Petty et al., 1985) despite our experiments have been carried out in anesthetized female rats.

Although it has previously been shown that estrogen is an important inducer of oxytocin receptors in several tissues (Zingg et al., 1995; Zhou and Forsling, 2000; Narita and Ichimaru, 2014; Narita et al., 2016), the current study does not show any difference in the change of IP evoked by oxytocin in control and ovariectomized rats. Because of that, topical administration of oxytocin onto the urinary bladder of ovariectomized rats was not performed. Plasma estrogen was not measured in the present study, which is a limitation of the present study, nevertheless, it would be expected that the rats were at least in a hypoestrogenism condition after 21 days as it has been shown in earlier studies (Deer and Stallone, 2016).

In the present study, oxytocin effects were also evaluated by *in situ* administration onto the urinary bladder in order to understand if the responses by intravenous injection were due to direct action on the detrusor muscle or through central mechanisms leading to activation of the autonomic nervous system. Topical (*in situ*) administration of oxytocin onto the outside of the urinary bladder showed a similar effect compared to the intravenous injection. This finding suggests that oxytocin can diffuse the serosa layer of the bladder and act directly on receptors in detrusor layer when it is topically administrated. Oxytocin evoked a peak drop in IP ∼5 min after topical administration, whereas intravenous injection of oxytocin elicited the peak decrease in IP ∼15 min after starting the infusion. The drop in IP either after intravenous injection or *in situ* administration of oxytocin lasted for additional 5 min after achieving the peak response. Those differences in the latency for the onset of fall in IP likely lay in the fact that oxytocin after topical application can achieve the receptors in the bladder faster than after intravenous injection. In the last one, oxytocin is dissolved in the circulation and needs to reach out the bladder in order to bind to the receptors, taking a while in order to do that.

Our findings also demonstrated that intravenous injection of oxytocin receptor antagonist significantly attenuated the drop in IP elicited either by intravenous or *in situ* oxytocin, suggesting that oxytocin is acting in its receptors localized in the urinary bladder.

While OT is certainly most known to contract smooth muscle (such as the uterus), relaxatory effects are not unheard of. Studies of Duridanova et al. (1997) have shown that the oxytocin-related relaxation may result from the activation of Ca^2+^-sensitive K^+^ conductivity via activation of IP3-induced release of Ca^2+^ from the submembrane located in the cisternae of the sarcoplasmic reticulum Ca^2+^ stores and in turn, this evokes a non-inactivating component of K^+^ channels, hyperpolarizing the cell membrane.

The release of oxytocin in the circulation has been demonstrated in rats during conditioned fear stimuli in a pathway which involves the A2 noradrenergic neurons in the medulla oblongata (Zhu and Onaka, 2002). Furthermore, oxytocin can be released after noxious stimuli mediated by A1 noradrenergic neurons in the medulla (Onaka et al., 2001). Indeed, it is possible that physiologically, the release of oxytocin and its action in the urinary bladder can be important to avoid the micturition or urine loss during stressful conditions as fear and pain. Nevertheless, it is unknown if the amount of oxytocin varies under different stress conditions and also if oxytocin at high concentration could also bind to vasopressin receptors, eliciting a bladder overactivity (Smith et al., 2011; Hattori et al., 2019).

In conclusion, the current findings suggest that (1) intravenous oxytocin decreases IP due to bladder relaxation not dependent on changes in cardiovascular parameters, (2) *in situ* topically applied oxytocin also decreases IP and has local bladder effect, (3) oxytocin binds directly in receptors located in the urinary bladder and the effect of OT on the bladder does not appear to be affected by the plasma estrogen levels in female rats, as suggested by the absence of change due to ovariectomy.

## Data Availability Statement

The datasets generated for this study are available on request to the corresponding author.

## Ethics Statement

The animal study was reviewed and approved by the Ethics Committee for Animal experiments [Comissão de Ética no Uso de Animais (CEUA)] at Faculdade de Medicina do ABC (FMABC), Centro Universitário Saúde ABC. Protocol number 007/2011.

## Author Contributions

EC, LS, and LA have carried out the functional experiments. EC, IR, JS, RM, and GG were responsible for the qPCR experiments. EC, BA, DV, and PA have worked on data analysis and discussion. MS has designed the experiments, performed the statistical analysis, obtained the research grant, and also have written the manuscript with EC and PA. All the authors equally contributed to development of the manuscript.

## Conflict of Interest

The authors declare that the research was conducted in the absence of any commercial or financial relationships that could be construed as a potential conflict of interest.
